# Supplementary figures and images for: Identification of Novel Modulators of the ALT Pathway Through a Native FISH-Based Optical Screen
Source: bioRxiv. 2024 Nov 15:2024.11.15.623791. Preprint. [Version 1] doi: 10.1101/2024.11.15.623791 (PMC11601530; doi:10.1101/2024.11.15.623791)

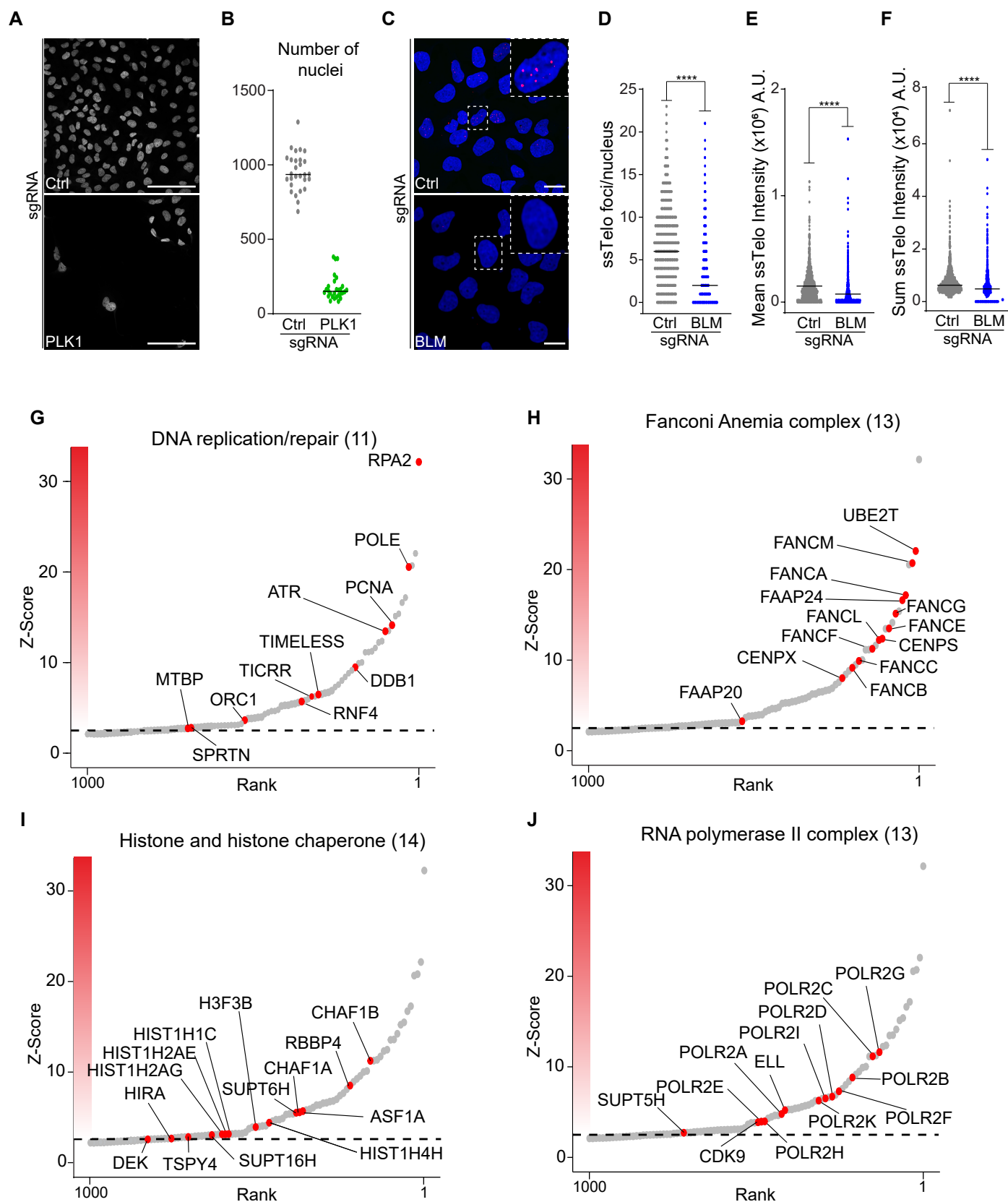

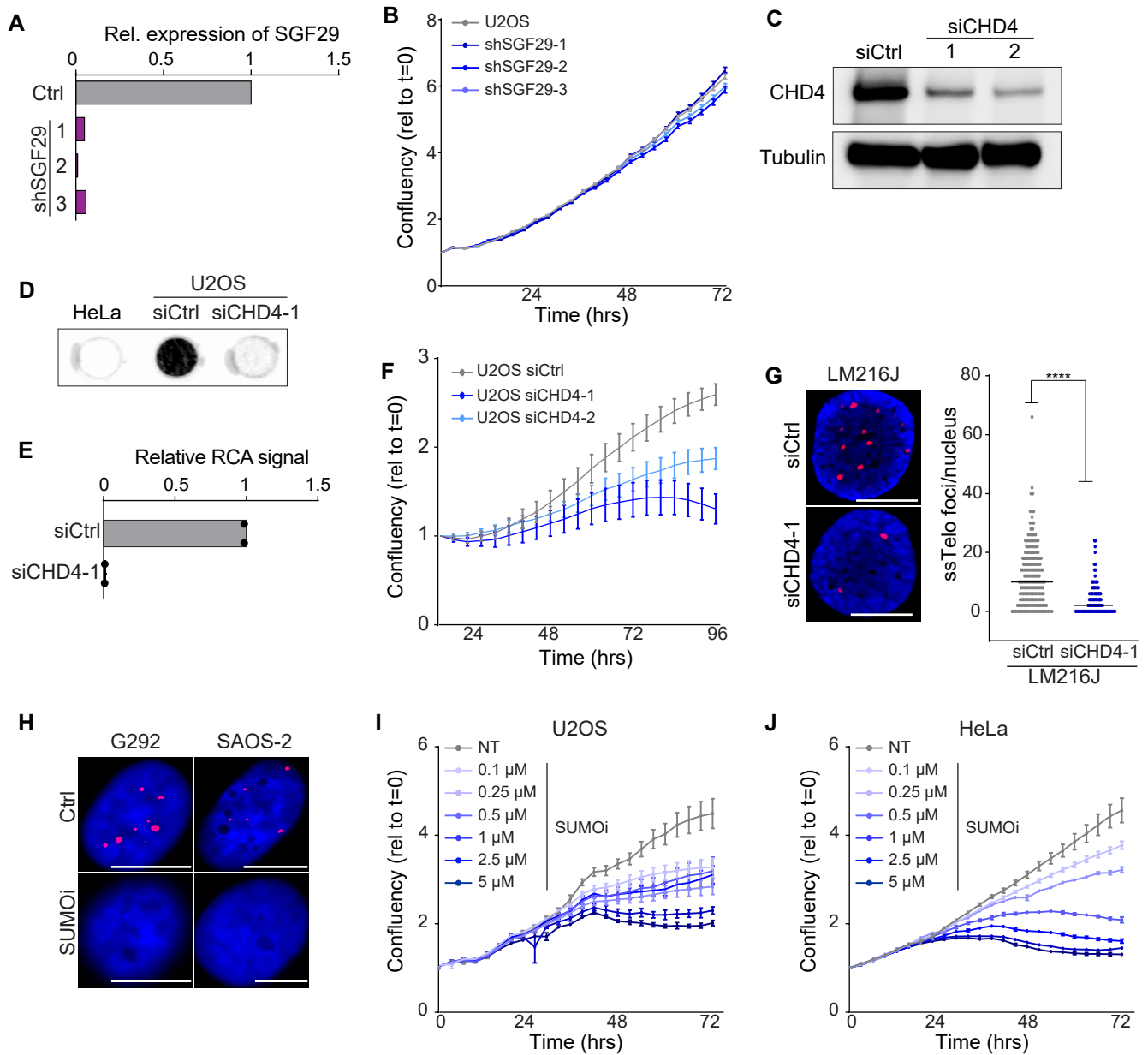

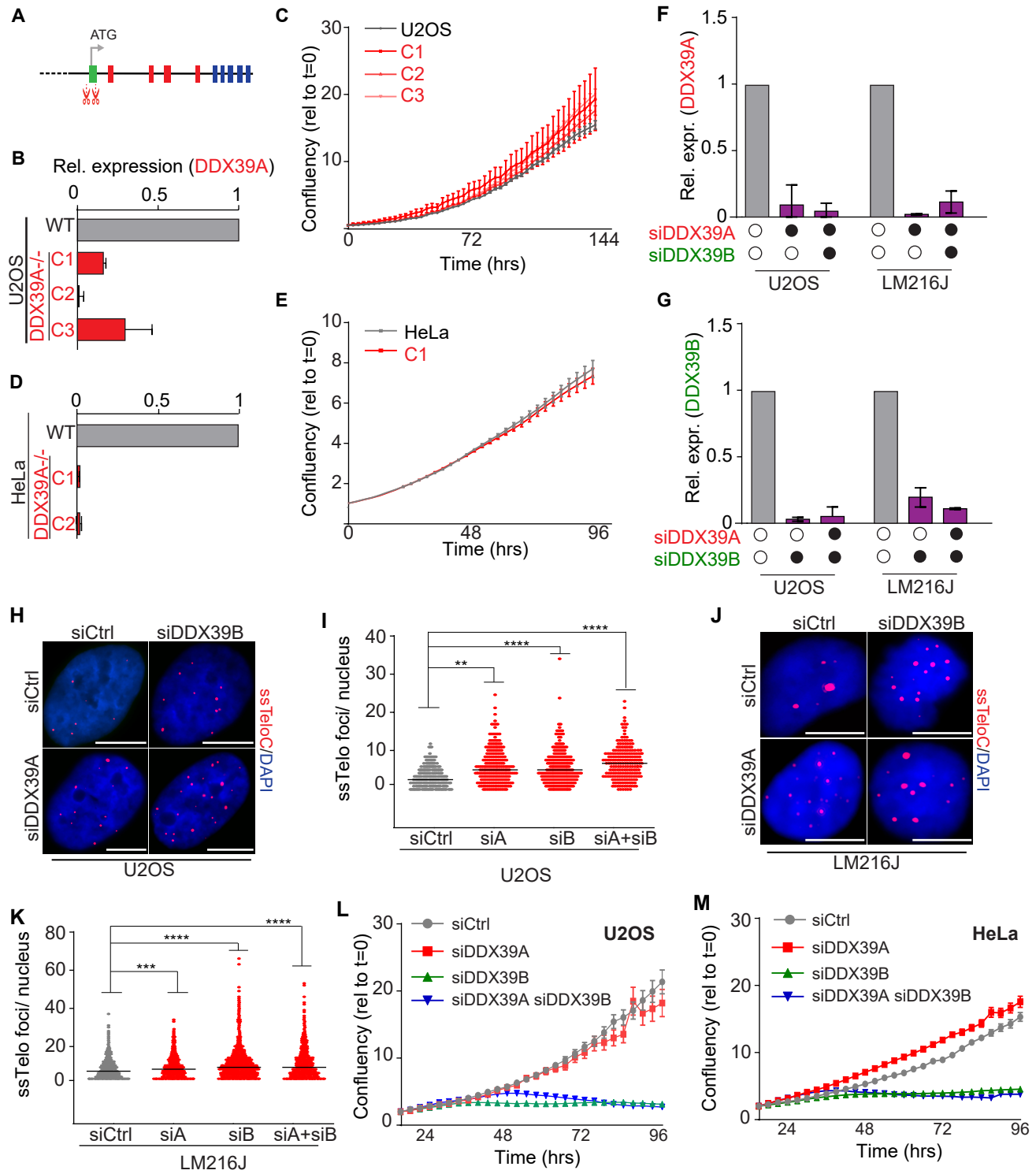

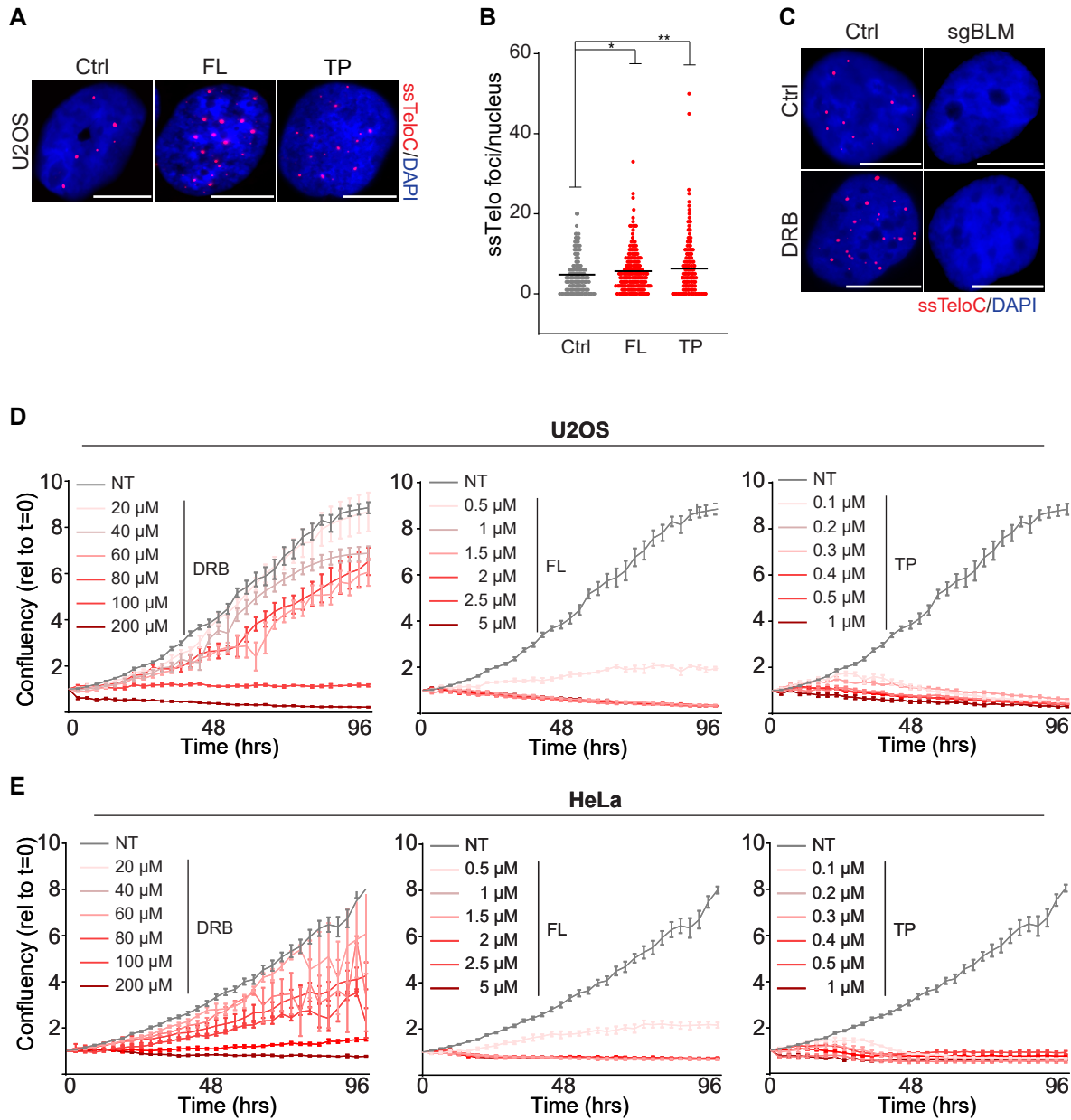

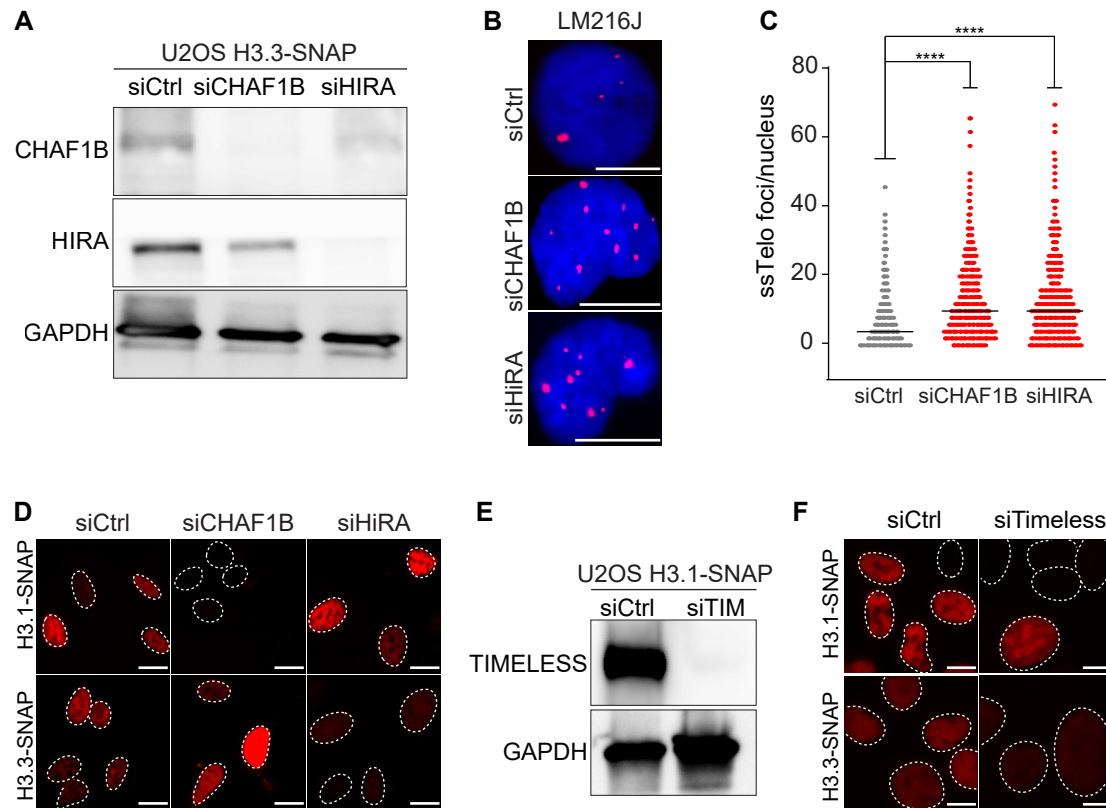

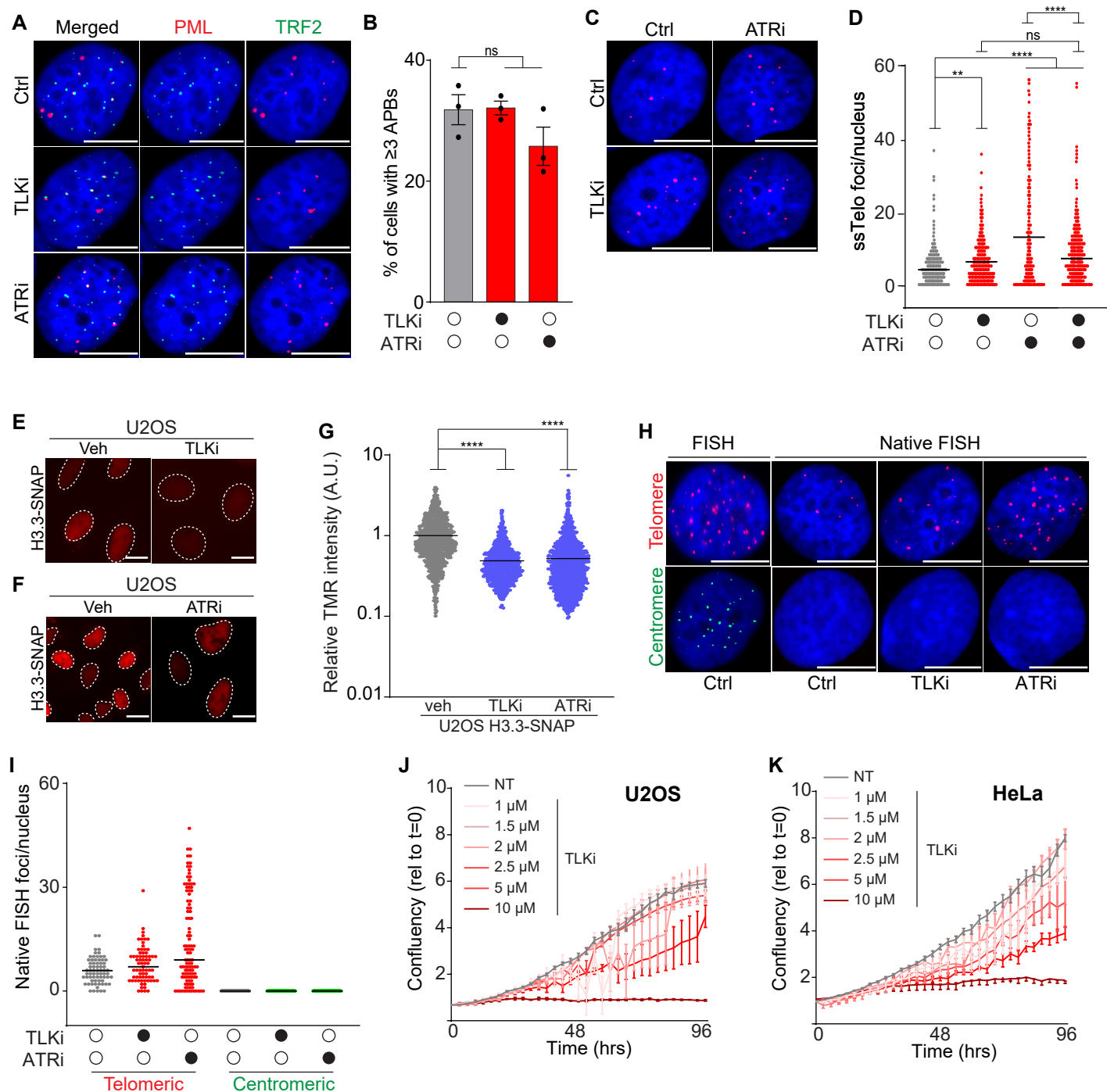

Supplement: 3 [file NIHPP2024.11.15.623791v1-supplement-3.pdf]
